# Early-Life Gut Microbiota Governs Susceptibility to Colitis via Microbial-Derived Ether Lipids

**DOI:** 10.34133/research.0037

**Published:** 2023-01-13

**Authors:** Yanjun Liu, Chunhua Jiao, Tao Zhang, Xue Li, Panpan Li, Meishan Lu, Zhan Ye, Yanpeng Du, Runfeng Du, Wenlong Zhang, Jie Xu, Zhaojun Zheng, Yongjiang Xu, Changhu Xue, Yi Zhang, Yuanfa Liu

**Affiliations:** ^1^ State Key Laboratory of Food Science and Technology, School of Food Science and Technology, Collaborative Innovation Center of Food Safety and Quality Control in Jiangsu Province, Jiangnan University, 1800 Lihu Road, Wuxi 214122, Jiangsu, China.; ^2^ College of Food Science and Engineering, Ocean University of China, 5 Yushan Road, Qingdao, 266003, China.; ^3^ Department of Colorectal Surgery, The First Affiliated Hospital of Nanjing Medical University, Nanjing 210000, China.; ^4^ Department of Gastroenterology, The First Affiliated Hospital of Nanjing Medical University, Nanjing 210000, China.; ^5^ College of Food Science and Engineering, Nanjing University of Finance and Economics, Nanjing 210023, Jiangsu, China.

## Abstract

Localized intestine inflammation could induce short-term increases in colonic oxygenation and leads to increases in the aerobic bacteria population and reduction in the anaerobic bacteria population by changing the intestinal environment. However, the mechanisms involved and the associated functions of intestinal anaerobes in gut health still remain unclear. Here, we found that early-life depletion of gut microbiota exacerbated later colitis, while mid-life microbiota depletion showed partially reduced colitis. Notably, we observed that early-life gut microbiota depletion confers susceptibility to ferroptosis in colitis. In contrast, restitution of early-life microbiota conferred protection against colitis and inhibited ferroptosis triggered by gut microbiota dysbiosis. Similarly, colonization with anaerobic microbiota from young mice suppressed colitis. These results may attribute to high abundance of plasmalogen-positive (plasmalogen synthase [PlsA/R]-positive) anaerobes and plasmalogens (one of the common ether lipids) in young mice but reduced abundance in the development of inflammatory bowel disease. Early-life anaerobic bacteria elimination also resulted in the aggravation of colitis, while this aggravation phenotype was reverted by plasmalogen administration. Interestingly, plasmalogens inhibited ferroptosis triggered by microbiota dysbiosis. We further find that the alkenyl-ether group of plasmalogens was critical to colitis prevention and ferroptosis inhibition. These data point to one of the mechanisms by which the gut microbiota controls susceptibility to colitis and ferroptosis early in life via microbial-derived ether lipids.

## Introduction

Inflammatory bowel disease (IBD) is a chronic and relapsing gastrointestinal disorder that presents intestinal inflammation, with growing incidence worldwide [1]. While the precise cause of IBDs is unknown, several factors have been implicated in the pathogenesis, including age, genetics, immune system, and environmental factors [2]. One accepted hypothesis of its pathogenesis is gut microbiota alteration [3]. Recent work highlighted that gut microbiota dysbiosis plays an essential role in the pathogenesis of IBD [4]. Besides the microbiota, previous studies demonstrated that environmental factors, including oxygen, osmolality, nitrogen, and pH in the gut, were involved in the regulation of IBD [5]. It is well known that a healthy gut is characterized by low oxygen levels and enriched communities of obligate anaerobes [4], while IBD is characterized by high contents of nitrogen and reactive oxygen species (ROS). Circumstantial evidence of a role for obligate anaerobes in IBD has been accumulating for years [4]. Intestinal inflammation could induce the increases in colonic oxygenation and imbalance of bacterial population structure, especially the aerobic bacteria population and anaerobic bacteria population. Mounting evidence showed the beneficial effects of obligate anaerobes and their products on the colonic environment in IBDs, including *Bifidobacterium* and *Clostridium butyricum* [1]. Notably, microbiota dysbiosis marked by an increase in facultative anaerobes and a reduction of obligate anaerobes has been observed in patients suffering from IBDs [6].

One of the most marked differences between obligate anaerobes and other organisms is the stark difference between aerobic and anaerobic biosynthetic routes of plasmalogen [5]. Plasmalogens, the most common form of ether lipids, are widely distributed in animals and anaerobic bacteria, sensitive to ROS, and involved in membrane structure, signaling and protection against ROS [7]. Although plasmalogen synthase (pls) has been observed to be widespread in many members of the gut microbiota, including obligate anaerobes and some facultative anaerobes [7], there is still relatively little information regarding the plasmalogen-producing bacterial in gut microbiota. Besides, plasmalogens have not been found in aerobic and facultative anaerobic bacteria until now. As such, a better understanding of and in-depth characterization of bacterial communities of plasmalogen-positive species (pls [PlsA/R]-positive) and their role in gut inflammatory diseases is needed to define therapeutic targets. Although studies have documented that gut microbiota plays a crucial role in intestinal health and disease, there is still relatively little information regarding age-related changes in gut microbiome from young adulthood to mature adult stage in mice and the mechanisms underpinning the ability of the gut microbiota on intestinal health, particularly in early life. Of note, ether-linked phospholipids have been revealed to be critical in driving ferroptosis, which is a regulator of intestinal diseases [8]. In this study, we sought to address the role of the microbiota harboring the anaerobic plasmalogen biosynthetic pathway in establishing intestinal homeostasis in early life. We hypothesized that plasmalogen-positive (pls [PlsA/R]-positive) bacterial species and plasmalogen conferred beneficial effects on intestine homeostasis in early life.

## Results

### Early-life microbiota depletion exacerbates colitis

**Fig. 1.**
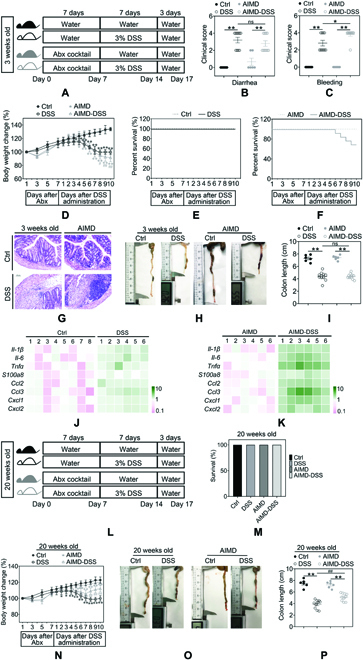
Early-life but not mid-life antibiotic-induced microbiota depletion exacerbates DSS-induced colitis. (A) Study design using male AIMD mice at the age of 3 weeks. Three-week-old mice were subjected to a 1-week oral administration of a broad-spectrum antibiotic cocktail and then treated with 3% DSS for 7 days. (B and C) Stool consistency and colorectal bleeding were scored in antibiotic-treated or untreated mice at the age of 3 weeks. (D) Body weight of antibiotic-treated or untreated mice at the age of 3 weeks was measured daily. (E and F) Survival curve of antibiotic-treated or untreated mice at the age of 3 weeks following treatment with 3% DSS. (G) Colon tissues of antibiotic-treated or untreated mice at the age of 3 weeks were examined histologically after H&E staining. Scale bars, 100 μm. (H and I) Antibiotic-treated or untreated mice at the age of 3 weeks were euthanized on day 17, and colon lengths were measured. (J and K) Heatmap showing the relative expression levels of genes involved in the inflammatory response in antibiotic-treated or untreated mice at the age of 3 weeks. (L) Study design using male AIMD mice at the age of 20 weeks. Twenty-week-old mice were subjected to a 1-week oral administration of a broad-spectrum antibiotic cocktail and then treated with 3% DSS for 7 days. (M) Survival curve of antibiotic-treated or untreated mice at the age of 20 weeks following treatment with 3% DSS. Stool consistency and colorectal bleeding were scored. (N) Body weight was measured daily. (O and P) Antibiotic-treated or untreated mice at the age of 20 weeks were euthanized on day 17, and colon lengths were measured. Data are shown as individual points with mean ± SEM. From (B) to (K), *n* = 6 mice at the age of 3 weeks (Ctrl and AIMD group), *n* = 10 mice (DSS and AIMD-DSS group). (L to P) *n* = 6 mice at the age of 20 weeks (Ctrl and AIMD group), *n* = 10 mice (DSS and AIMD-DSS group). ns, no significant difference, *P < 0.05 and **P < 0.01.

In the present study, to confirm whether the age-associated alteration of gut microbiota composition between young and adult mice is involved in the pathogenesis of colitis, we used a broad-spectrum antibiotic cocktail to deplete the gut microbiota (Fig. 1A and L) in 3-week-old (thereafter referred to as young mice) and 20-week-old mice (thereafter referred to as mature adult mice). We then checked the impact of gut microbiota depletion in different stages of life on intestinal health. Culture-dependent (Fig. S1A) and culture-independent analysis (Fig. S1B) confirmed that the fecal microbiota was fully depleted in antibiotic-induced microbiota-depleted (AIMD) mice. In agreement with previously published reports [9], early-life antibiotic-induced microbial depletion leads to further exacerbated colitis in mice after dextran sulfate sodium (DSS) challenge, as indicated by increased disease activity index score (stool consistency and colorectal bleeding, Fig. 1B and C), aggravated body weight loss (Fig. 1D), increased mortality (Fig. 1E and F), and colon shortening (Fig. 1H and I). Moreover, colon of antibiotic cocktail-treated mice exhibited loss of crypts and extensive ulceration after DSS exposure (Fig. 1G). Consistent with those results, antibiotic-induced microbiota depletion upregulated mRNA levels of inflammatory cytokines (*IL-1β*, *Tnf-α*, and *IL-6*) and chemokines (*Cxcl1*, *Cxcl1*, *Ccl2*, and *Ccl3*), except S100a8, as compared with DSS-treated mice (Fig. 1J and K). However, inconsistent with young mice, mid-life microbiota depletion (Fig. 1L) reduced colitis upon DSS challenge (Fig. 1M to P). Therefore, these findings showed a contrary pattern of susceptibility from early-life and midlife microbial dysbiosis on colitis, probably reflecting the potential role of gut microbiota alteration mediated by age changes in intestinal health.

### Early-life microbiota confers susceptibility to ferroptosis in colitis

**Fig. 2.**
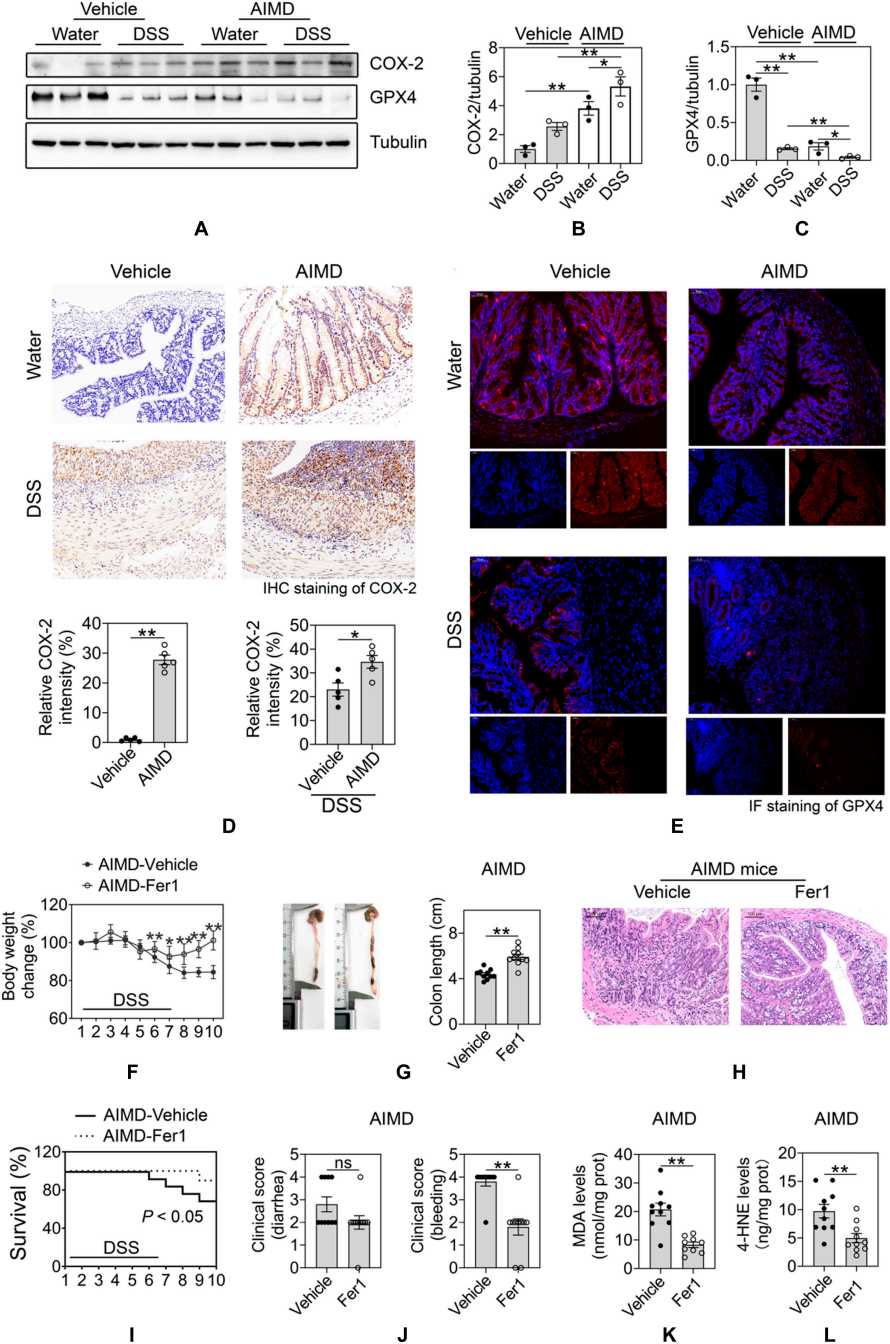
Early-life microbiota confers susceptibility to ferroptosis in colitis. (A to C) Representative immunoblots and quantitative analysis of ferroptosis markers COX-2, GPX4. (D) Representative immunohistochemistry (IHC) staining and quantitative analysis of COX-2. (E) Representative immunofluorescent (IF) staining of GPX4. Its immunofluorescence analysis was shown in Fig. S4A and B. Body weight of antibiotic-treated mice after treatment of Fer1 and 3% DSS. (G) Antibiotic-treated mice after treatment of Fer1 and 3% DSS were euthanized, and colon lengths were measured. (H) Colon tissues of antibiotic-treated mice after treatment of Fer1 and 3% DSS were examined histologically after H&E staining. Scale bars, 100 μm. (I) Survival curve of antibiotic-treated mice after treatment of Fer1 and 3% DSS. (J) Stool consistency and colorectal bleeding were scored. (K and L) Colonic MDA levels and 4-HNE levels were measured. Data are shown as individual points with mean ± SEM. From (A) to (C), *n* = 3 mice, (D and E), *n* = 5 mice, (F to L), *n* = 10 mice. ns, no significant difference, *P < 0.05 and **P < 0.01.

Ferroptosis was identified as a cause of colitis-associated cell death of intestinal epithelial cells (IECs) [10], one of the characteristics of IBD [11]. Thus, we hypothesized that regulation of ferroptosis may be an important process in maintaining intestinal homeostasis. In our present study, we observed that DSS-induced colitis (Fig. S2A, B, and E) was accompanied by enhanced colonic iron (Fig. S2C), malondialdehyde (MDA) levels (Fig. S2D), and COX-2 (cyclooxygenase-2) signals, and reduced GPX4 (glutathione peroxidase 4) levels (Fig. S2F to P) in the colon tissues. These results suggested that ferroptosis was induced in the colonic IECs of colitis, a finding consistent with the previous study. To confirm whether ferroptosis is involved in the enhanced susceptibility from early-life microbial dysbiosis to colitis, we then measured COX-2, GPX4 levels, and iron contents in the colonic tissues of AIMD mice. Immunohistochemistry and immunofluorescent assay of ferroptosis biomarkers revealed elevated positive signals for COX-2 and reduced GPX4 signals in the epithelial cells from mice with early-life microbial dysbiosis. We also found that colonic MDA and 4-hydroxynonenal (4-HNE) levels were obviously increased in mice with early-life microbial dysbiosis (Fig. S3). Of note, after 1 week of DSS exposure, AIMD mice exhibited high ferroptosis susceptibility as evidenced by increased lipid peroxide levels, elevated COX-2-positive signals, and reduced GPX4-positive signals (Fig. 2A to E) and Fig. S4A and B). Furthermore, ferroptosis inhibitor ferrostatin-1 (Fer1) extended mice survival and protect IECs against colitis in AIMD mice (Fig. 2F to I) through inhibition of lipid peroxide accumulation (Fig. 2J to L), which suggested that blockage of ferroptosis rescued colitis aggravated by early-life microbial dysbiosis. Taken together, the data presented above indicated a potential nexus between ferroptosis and microbial dysbiosis in colitis.

### Intestinal microbiota in early life contributes to suppression of colitis and ferroptosis

**Fig. 3.**
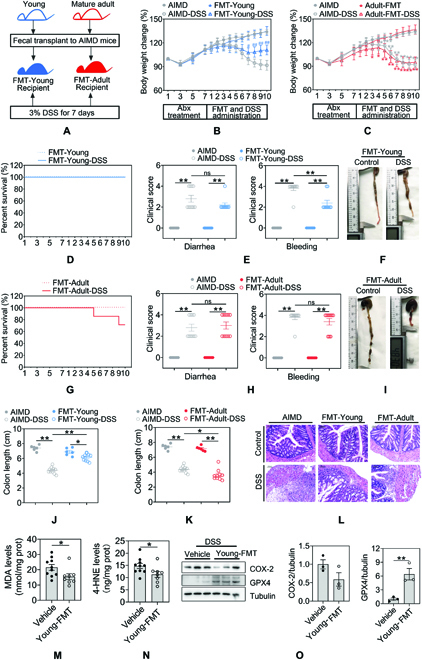
Intestinal microbiota in early life via FMT contributes to the ability to fight DSS-induced colitis and ferroptosis. (A) Study design using FMT. Young normobiotic mice 3 weeks old were treated with a broad-spectrum antibiotics cocktail, followed by 3% DSS for 7 days and fecal microbiota transfer to ensure gut microbiota colonization from 3-week-old or 20-week-old donor mice. (B and C) The body weight of mice received FMT from young donors Young-FMT or adult donors Adult-FMT was measured daily. (D and G) Survival curve of mice received FMT either from young donors or adult donors following treatment with 3% DSS. (E and H) Stool consistency and colorectal bleeding were scored in mice received FMT from young donors or adult donors. (F and J) Mice received FMT from young donors were euthanized, and colon lengths were measured. (I and K) Mice received FMT from adult donors were euthanized, and colon lengths were measured. (L) Colon tissues of mice received FMT from young donors or adult donors were examined histologically after H&E staining. Scale bars, 100 μm. (M and N) Colonic MDA levels and 4-HNE levels were measured in DSS-treated mice received FMT from Young donors. (O) Representative immunoblots and quantitative analysis of ferroptosis markers COX-2, GPX4. Data are shown as individual points with mean ± SEM. From (A) to (N), *n* = 6 to 10 mice, (O), *n* = 3 mice. ns, no significant difference, *P < 0.05 and **P < 0.01.

**Fig. 4.**
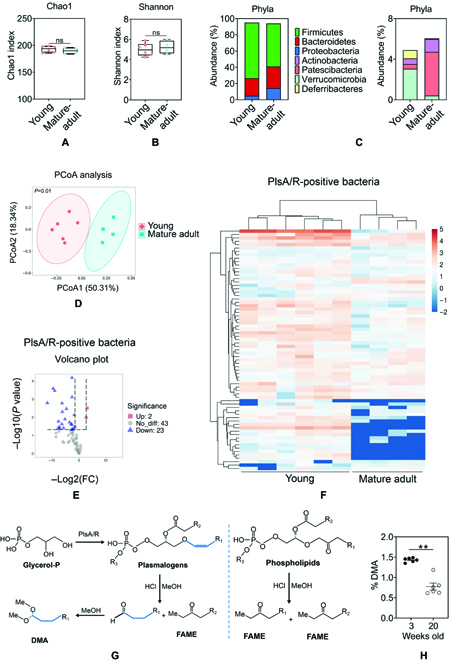
Plasmalogen-positive species are enriched in the early-life gut microbiome of mice. Colon contents of 3-week-old and 20-week-old mice were collected, and its microbiota profiles were characterized via 16S rRNA gene sequencing. (A and B) Comparisons of α-diversity of gut microbiota composition between 3-week-old and 20-week-old mice as assessed by the Chao1 and Shannon index. (C) The gut bacterial composition at the phylum level in 3-week-old and 20-week-old mice. (D) Principal coordinate analysis of gut microbiota by unweighted and weighted UniFrac between 3-week-old and 20-week-old mice. Each symbol indicates 1 mouse. *p* = 0.01, significant difference between 3-week-old and 20-week-old mice. (E and F) A volcanic plot and heatmap depicting the differential plasmalogen-positive bacterial species between 3-week-old and 20-week-old mice. Red dots, significantly up-regulated species. Blue dots, significantly down-regulated species. Grey dots, no significant difference. (G) The schematic of a proposed pathway for anaerobic synthesis of plasmalogens, followed by conversion of plasmalogens to dimethyl-acetal (DMA) in the presense of acid/methanol. Fatty acid methyl ester (FAME) was formed from fatty acid from sn-1 and sn-2 positions of diacylglycerophospholipids and the sn-2 position of plasmalogens. (H) The abundance of DMA in colon contents of 3-week-old and 20-week-old mice. Data are shown as individual points with mean ± SEM. From (A) to (E), *n* = 6 mice (Young) and *n* = 4 mice (Mature-adult); (G) *n* = 6 mice per group. ns, no significant difference, *P < 0.05 and **P < 0.01.

To further explore the process in that early-life microbiota depletion renders mice susceptible to ferroptosis, we sought to test whether microbiota from young donor mice or adult donor mice in AIMD recipient mice alters the susceptibility to colitis and ferroptosis. According to the scheme described in Fig. 3A, young normobiotic mice (3-week-old) were treated with broad-spectrum antibiotics, followed by administration of 3% DSS for 7 days and fecal microbiota transplantation (FMT) to ensure gut microbiota colonization from either the young- (3-week-old) or adult (20-week-old)-donor mice. Of note, restoring gut microbiota from healthy young mice reverted clinical consequences of colitis and indicators of intestinal inflammation significantly, while the overall response of FMT-Adult-treated mice was comparable to AIMD mice in colitis (Fig. 3). When compared with control mice, transplantation of gut bacteria from young mice showed reduced signs of intestinal inflammation, as indicated by milder body weight loss (Fig. 3B), extended survival (Fig. 3D), reduction of the pathological score (Fig. 3E), and counteraction of colon shortening (Fig. 3F and J), while FMT from adult mice resulted in high susceptibility to colitis, as indicated by significant body weight loss (Fig. 3C), increased mortality (Fig. 3G), increased clinical score (Fig. 3H), and shortening of the colon (Fig. 3I and K). Similarly, transplantation of gut bacteria from young mice, but not adult mice, exhibited the improvement of extensive ulceration, loss of crypts, and inflammation after DSS exposure (Fig. 3L). On the contrary, gut microbiota colonization from adult (20-week-old) donor mice had no overt effect on intestinal inflammation in AIMD mice during experimental colitis, as indicated by comparable ulceration, loss of crypts, and inflammation (Fig. 3L). Thus, these results demonstrate that colonization with early-life microbiota counteracts intestinal inflammation caused by the DSS challenge. In addition, transplantation of gut bacteria from young mice significantly reduced colonic MDA levels (Fig. 3M) and 4-HNE (Fig. 3N) levels compared with normal mice. COX-2 protein expression was significantly suppressed, and GPX4 expression was increased after transplantation of gut bacteria from young mice (Fig. 3O). These results indicate that early-life gut microbiota colonization prevents ferroptosis through the regulation of ferroptosis-related proteins.

### Plasmalogen-positive species are enriched in the early-life human gut microbiome and reduced in the development of colitis

To validate our observations above, we here determined whether gut microbiota, represented by the bacteria residing in colonic contents, differed between young and mature adult male mice. Mounting evidence suggests that colitis-associated microbiota shifts were strongly involved in the development of colitis, thereby regulating gut homeostasis [1]. We first characterized microbiota profiles of 3-week-old and 20-week-old mice in the colon contents. We performed 16S ribosomal RNA (rRNA) gene sequencing on the colon contents (Fig. 4). Analysis of bacterial communities showed no significant differences in alpha diversity (Fig. 4A and B) and beta-diversity (Fig. 4D) analysis metrics between young and adults mice, in line with previous reports [5]. However, microbiome analysis revealed distinct differences in the gut microbiome between young and mature adult mice (Fig. 4C and D), represented by the abundance of bacteria from the phyla and principal coordinate analysis. Plasmalogens, the most common form of ether lipids in mammals and anaerobe, contain a vinyl ether-linked fatty alcohol at position sn-1 and has been reported to be essential components for governing ferroptosis [8]. Interestingly, we further confirmed that fecal ether lipids levels, including plasmalogens and ether-linked phospholipids, were remarkably reduced in mice with microbiota depletion, which suggested that those ether lipids could be microbial-derived (Fig. S5A and B). Here, on the basis of the biosynthetic characteristics of plasmalogen in anaerobe, we tried to address the role of plasmalogen-positive bacterial species in establishing intestinal homeostasis in early life.

It has been previously shown that the genes from microbiomes that contained the pls operon (encodes PlsA and PlsR proteins) were classified as a species as plasmalogen-positive, and the distribution of the pls operon helps us to assess the distribution of plasmalogen biosynthesis genes [7]. Notably, among the plasmalogen-related Pfams and plasmalogen-positive species identified by Jackson et al. [7], we observed the decreased distribution of plasmalogen-positive species in mature adult mice (Fig. 4E) compared to young mice as indicated by the volcano plot. On the basis of a combination of prior studies [7,12], we illustrate a schematic of proposed anaerobic synthesis pathway of plasmalogens, followed by conversion of plasmalogens to dimethyl-acetal (DMA) in the presence of acid/methanol (Fig. 4F) [5]. DMA was formed from aliphatic aldehyde groups from the sn-1 position of plasmalogens, while fatty acid methyl ester (FAME) was formed from fatty acid in diacylglycerophospholipids. We analyzed the profile and abundance of DMA derivatives (percentage relative to total FAME and DMA) that represent aliphatic chains from ether lipids (Fig. 4G). DMA was identified with the domination of C18:1 DMA, C18:0 DMA, C16:0 DMA, C12:0 DMA, and C11:0 DMA in the colon contents of 3-week-old and 20-week-old mice (Table S1). As detection of DMA abundance (% in DMA and FAME) in the colon contents of young mice was significantly higher (Fig. 4H) than in the adult mice (*P* < 0.01), this suggested the number of plasmalogens from the gut flora was significantly decreased in adult mice, compared with young mice. Of note, as detection of DMA levels in the colon contents of AIMD mice and fecal ether-linked phospholipids levels were significantly lower than in the specific pathogen-free (SPF) control mice (Fig. S6A and B), which underscored that these ether lipids were microbiota-dependent. On the basis of the reduction of plasmalogen-producing anaerobic bacteria and its plasmalogen levels in the colon contents of adult mice, these results indicate that plasmalogen-positive microbiota and its crucial metabolite (plasmalogens) decreased significantly with age, but whether this affects intestinal health was not known.

**Fig. 5.**
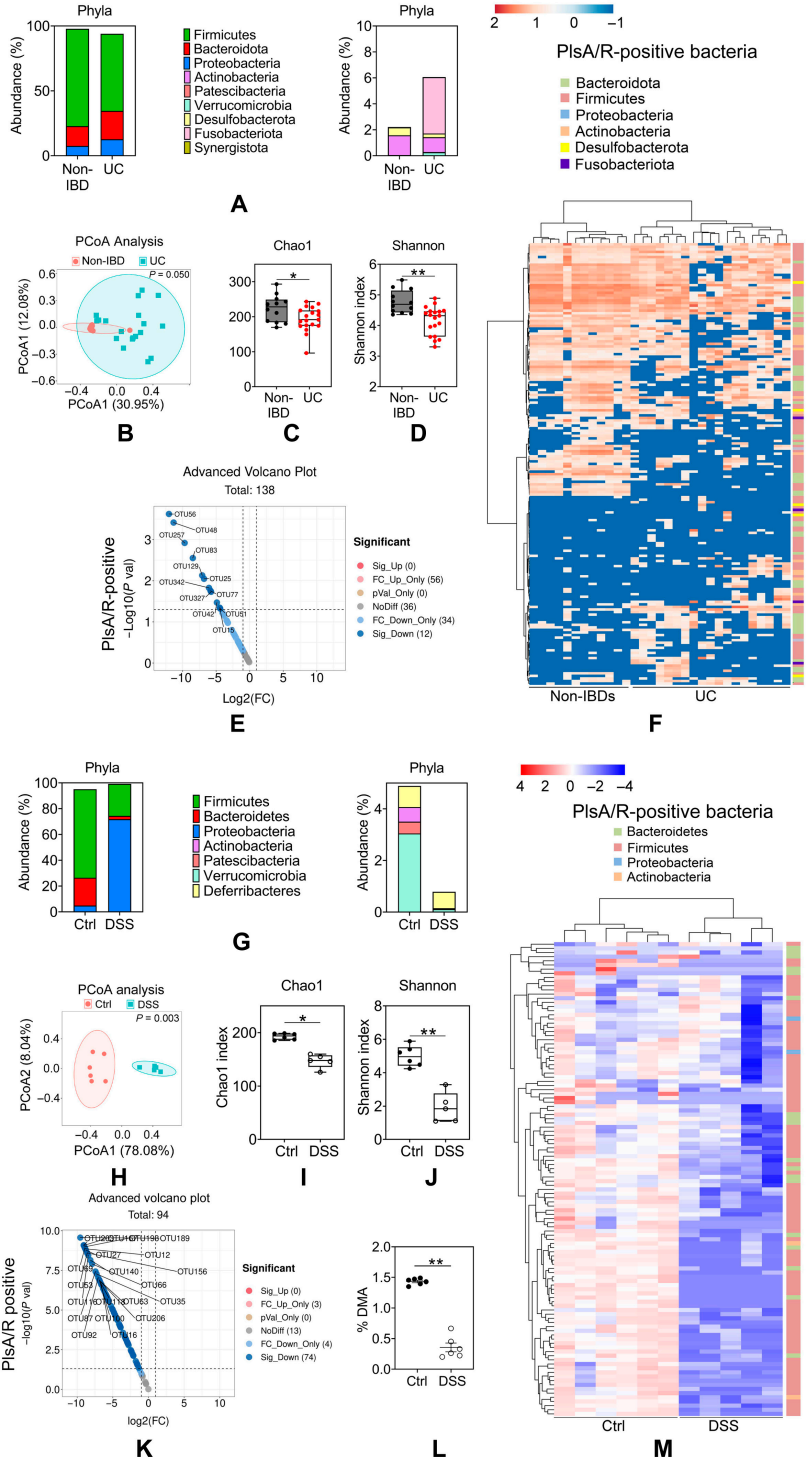
Reduction of plasmalogens-positive microbiota occurs in colitis. Non-IBD (*n* = 12) and ulcerative colitis (UC) (*n* = 19) stool samples were collected, and its microbiota profiles were characterized via 16S rRNA gene sequencing. In addition, colon contents of control and DSS-induced colitis mice were collected, and its microbiota profiles were characterized via 16S rRNA gene sequencing. (A) The gut bacterial composition at the phylum level in non-IBD and ulcerative colitis. (B) Principal coordinate analysis of gut microbiota by unweighted and weighted UniFrac between non-IBD and ulcerative colitis. Each symbol indicates 1 patient. P = 0.050, significant difference between non-IBD and ulcerative colitis. (C and D) Comparisons of α-diversity of gut microbiota composition between non-IBD and ulcerative colitis as assessed by the Chao1 and Shannon index. (E and F) A volcanic plot and heatmap depicting the differential plasmalogen-positive bacterial species between non-IBD and ulcerative colitis. Dark red dots, significantly up-regulated species. Dark blue dots, significantly down-regulated species. Gray dots, no significant difference. (G) The gut bacterial composition at the phylum level in control and DSS-induced colitis mice. (H) Principal coordinate analysis of gut microbiota by unweighted and weighted UniFrac between control and DSS-induced colitis. Each symbol indicates 1 mouse. *P* = 0.003, significant difference between control and DSS-induced colitis mice. (I and J) Comparisons of α-diversity of gut microbiota composition between control and DSS-induced colitis mice as assessed by the Chao1 and Shannon index. (K and M) A volcanic plot and heatmap depicting the differential plasmalogen-positive bacterial species between control and DSS-induced colitis mice. Dark red dots, significantly up-regulated species. Dark blue dots, significantly down-regulated species. Gray dots, no significant difference. (L) The abundance of DMA in colon contents of control and DSS-induced colitis mice. Data are shown as individual points with mean ± SEM. From (A) to (E), *n* = 12, Non-IBD; *n* = 19 ulcerative colitis, (F to M), *n* = 6 mice per group. ns, no significant difference, *P < 0.05 and **P < 0.01.

Our observation that colonic early-life microbiota, which is identified to be enriched with plasmalogen-positive species, contribute to the prevention of colitis development and driving ferroptosis susceptibility prompted us to test whether plasmalogen-positive microbiota plays a role in the development of colitis. Herein, we sought to investigate whether the reduced abundance of plasmalogen-positive bacteria is linked to aggravated colitis. We first characterized fecal microbiota profiles of non-IBD individuals (*n* = 12) aged 13 to 29 years and IBD patients (*n* = 19) aged 13 to 30 years, which showed significant separation of microbial profiles between patients with healthy and IBD individuals (Fig. 5A to D). Previous studies have shown that colon inflammation could induce proportional changes in the gut microbiota [13]. The above results also suggest that the microbial composition of feces from IBD patients is quite different from that of feces from the healthy population. Next, we analyzed the differences in plasmalogen-positive species between IBD patients and the healthy population. Jackson et al. have classified plasmalogen-positive species on the basis of the Pfam domains related to pls (PlsA/R) [7]. Our study assessed the OTU (operational taxonomic unit) levels of plasmalogen-positive bacteria containing plasmalogen biosynthesis genes and found that the abundance of plasmalogen-positive bacteria in IBD patients was significantly lower than in healthy people (Fig. 5E)). We next characterized microbiota profiles of DSS-induced colitis in the colon contents of young mice (3 weeks old) via 16S rRNA gene sequencing. Microbiome analysis revealed distinct differences in the gut microbiome between control mice and DSS-induced colitis mice, represented by the abundance of bacteria from the phyla (Fig. 5F)) and principal coordinate analysis (Fig. 5G). Assessment of bacterial communities showed a significant decrease in alpha diversity (Fig. 5H and I) analysis metrics between young and adult mice. Of note, we also observed the decreased distribution of plasmalogen-positive bacteria species in mice with colitis (Fig. 5J) when compared to SPF control mice. We then analyzed the distribution of DMA derivatives that represent aliphatic chains from plasmalogens (Fig. 5K). We found that DMA levels in the colon contents of DSS-treated mice were significantly lower than SPF control mice, which suggested that plasmalogens and plasmalogen-producing bacteria are negatively correlated with colitis.

### Intestinal anaerobic bacteria in early life protects against colitis

Accumulating evidence suggests that plasmalogens could be produced by anaerobic bacteria [7]. Therefore, to determine whether plasmalogens from the colon contents were secreted by anaerobic bacteria, we treated the mice at 3 weeks of age with metronidazole (Metro, one of the antibiotics with anaerobic bacteria-specific inhibition) for 1 week. The culture-dependent analysis confirmed that the anaerobic bacteria were depleted in the colon contents of metronidazole-treated mice (Fig. S1A). As detection of DMA levels in the colon contents of metronidazole-induced anaerobic bacteria-eliminated mice was significantly lower than the SPF control mice (Fig. S6), which underscored that anaerobic bacterium are the mainly microbial origin for the colonic plasmalogens in mice. We next treated metronidazole-induced anaerobic bacteria-eliminated mice with 3% DSS (Fig. S7A). Of note, we found that depletion of anaerobic bacteria in early life by metronidazole also leads to further exacerbated DSS-induced colitis in mice, as indicated by aggravated body weight loss (Fig. S7D), increased mortality (Fig. S7E and F), destruction of the epithelial architecture (Fig. S7G), and upregulated mRNA levels of inflammatory cytokines (*Tnf-α*, *IL-6*, and *IL-1β*) and chemokines (*Cxcl1*, *Ccl2*, and *Ccl3*) (Fig. S7J and K). Besides, depletion of anaerobic bacteria did not cause further aggravated clinical scores (Fig. S7B and C) and colon shortening (Fig. S7H and I).

Given that neither early-life microbiota depletion nor anaerobic bacteria elimination suppressed the development of acute colitis caused by DSS, we suggested that reduction of anaerobic bacteria in the early life of mice leads to a shift in their metabolites profile and ultimately does not show the protection against colitis. Thus, we hypothesized that the predominance of early-life anaerobic bacteria is required for the protective effects of colitis. To further support this hypothesis, we cultured the microbiota (Fig. S8A) under aerobic, microaerobic, or anaerobic conditions (referred to as Aero, MicroAero, and Anaero, respectively). AIMD mice were then colonized with the respective microbiota (Aero, MicroAero, and Anaero), and we treated the mice with 3% DSS for 7 days. The mice colonized with either aerobic or microaerobic microbiota developed severe and comparable intestinal inflammation in response to DSS exposure. In contrast, when compared with AIMD mice, mice colonized with anaerobic microbiota were protected against DSS-induced colitis, as indicated by reduced body weight loss (Fig. S8B), decreased clinical score (Fig. S8C and D), and counteraction of colon shortening (Fig. S8E and F). Moreover, histological examination of colon morphology revealed a marked decrease in the extent of ulceration and crypt damage in response to anaerobic microbiota colonization (Fig. S8G).

### Microbiota-derived plasmalogens suppress intestinal inflammation and ferroptosis

The above findings led us to hypothesize that enriched plasmalogens in the colonic microbiota environment are associated with decreased colitis susceptibility post early-life FMT. We next sought to address whether plasmalogen conferred beneficial effects on intestine homeostasis in early life. Thus, we treated a cohort of wild-type mice at 3 weeks of age with plasmenylethanolamine (PlsEtn, one of the ether lipids) through the oral route. PlsEtn-treated or vehicle-treated mice were fed with 3% DSS for 7 days (Fig. S9A). Interestingly, after DSS removal, PlsEtn treatment opposed body weight loss induced by DSS (Fig. S9B). Besides, PlsEtn treatment delayed colitis progression relative to vehicle-treated mice, as indicated by counteraction of colon shortening (Fig. S9C and D) and substantial decreases in the clinical score (Fig. S9E and F). Further, in line with these data, histological examination of colon morphology revealed reductions in the extent of ulceration, crypt damage, and submucosal infiltration of inflammatory cells in response to PlsEtn administration (Fig. S9G), indicating that the presence of PlsEtn in early life help to suppress colitis progression in mice. Notably, PlsEtn treatment not only suppressed colitis progression in young mice at 3 weeks of age but also in adult mice at 20 weeks of age (Fig. S10A to D).

Next, we evaluated the effect of PlsEtn on colitis and ferroptosis in metronidazole-induced anaerobic bacteria-eliminated mice at 3 weeks of age. Mice were firstly treated with metronidazole for 1 week, followed by administration of PlsEtn and DSS for 7 days through the oral route (Fig. S11A). Early-life anaerobic microbiota dysbiosis resulted in the aggravation of experimental colitis. However, PlsEtn supplementation rendered significant resistance against body weight loss (Fig. S9B), which suggested us that PlsEtn may participate in the regulation of anaerobic bacteria on colitis. Compared to the DSS- and metronidazole-treated mice, it extended the survival of mice (Fig. S11C) and opposed the shortening of colon length (Fig. S11F and G) and delayed colitis progression induced by DSS treatment, as indicated by the attenuation of the overall pathological score (Fig. S11D and E). In addition, PlsEtn treatment significantly decreases the mRNA expression of inflammatory cytokines (*Tnf-α*, *IL-6*, and *IL1β*) and chemokines (*Cxcl1*, *Cxcl1*, *Ccl2*, and *Ccl3*), except S100a8, as compared with PlsEtn-untreated mice (Fig. S11H). Hematoxylin and eosin (H&E) staining of colon morphology revealed a marked decrease in the extent of epithelial disruption and crypt damage (Fig. S11I) in the colon of PlsEtn-treated mice. Supporting the above data, we observed that PlsEtn treatment promoted M2 macrophage polarization in the colon (Fig. S11J and K), as indicated by staining for the M1 macrophage marker, iNos, and M2 macrophages marker, CD163. The above results demonstrated that PlsEtn treatment attenuated intestinal inflammation and promoted M1/M2 macrophages rebalance.

**Fig. 6.**
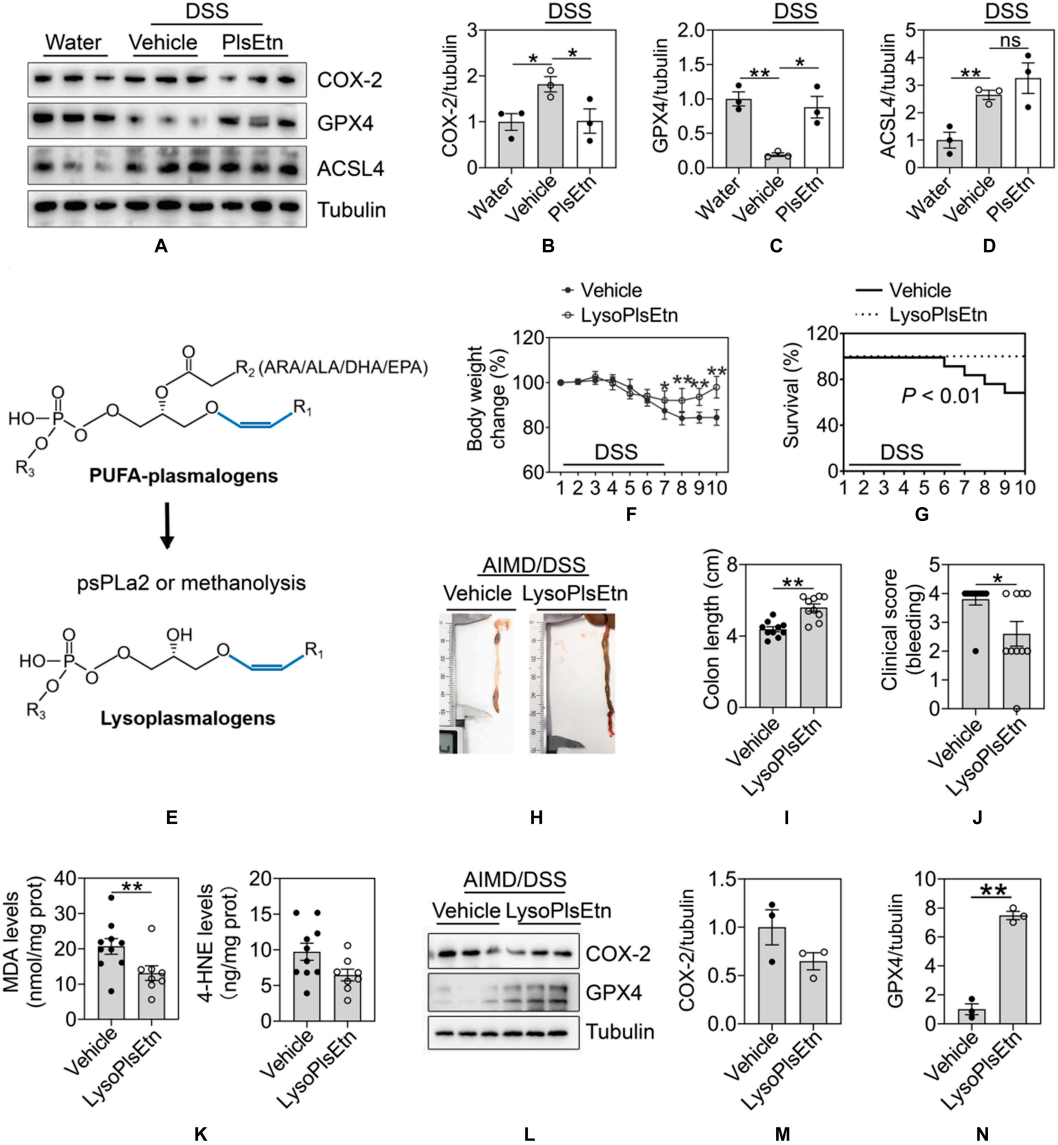
Alkenyl-ether group was critical to ferroptosis inhibition of plasmalogens. (A to D) Representative immunoblots and quantitative analysis of ferroptosis-related proteins (COX-2, GPX4, and ACSL4) in DSS-induced colitis mice after treatment of PlsEtn. (E) Enzyme catalyzing the degradation or methanolysis reaction from PUFA-Plasmalogens to lysoplasmenylethanolamine. Mice were treated with lysoplasmenylethanolamine and 3% DSS. (F) The body weight of mice received lysoplasmenylethanolamine was measured daily. (G) Survival curve of mice received lysoplasmenylethanolamine. (H and I) Mice received FMT from young donors were euthanized, and colon lengths were measured. (J) Colorectal bleeding were scored in mice received lysoplasmenylethanolamine. (K) Colonic MDA levels and 4-HNE levels were measured. (L to N) Representative immunoblots and quantitative analysis of ferroptosis markers (COX-2 and GPX4) in DSS-induced colitis mice after treatment of PlsEtn. From (A) to (C) and (L) to (N), *n* = 3, (F to K), *n* = 10 mice per group. ns, no significant difference, *P < 0.05 and **P < 0.01.

Notably, FAR1-TMEM189 mediated endogenous polyunsaturated fatty acid (PUFA)-plasmalogens synthesized in peroxisomes and endoplasmic reticulum could act as substrates for lipid peroxidation and then promote ferroptosis [14]. However, the effect of exogenous plasmalogens on ferroptosis has not been investigated. Of note, in colitis aggravated by early-life microbial dysbiosis, we found that the protein levels of colonic GPX4 and COX-2, well-accepted markers of ferroptosis, show significant differences after DSS treatment (Fig. 6A to D). Meanwhile, PlsEtn treatment can significantly increase GPX4 and decrease COX-2 (Fig. 6A to D). Plasmalogens synthesized in humans often contain PUFA at the sn-2 position of the glycerol moiety. In particular, iron oxidation of PUFAs and production of lipid hydroperoxides initiates ferroptosis. To test the role of the alkenyl-ether group at position sn-1 in ferroptosis, lysoplasmenylethanolamine (lysoPlsEtn) with a vinyl ether linkage was prepared from PlsEtn via methanolysis (Fig. 6). Indeed, lysoPlsEtn significantly alleviated arachidonic acid-induced HT29 cell damage (Fig. S12). These decreases of cell viability and cytotoxicity were partly abolished in cells exposed to the GPX4 inhibitor, ML210. Collectively, these results demonstrate that GPX4 inhibition is necessary for plasmalogens to protect against cell damage. We then performed a follow-up experiment in AIMD mice switching the PlsEtn treatment to lysoPlsEtn treatment, which revealed that lysoPlsEtn treatment resulted in reduced body weight loss (Fig. 6F), extended survival (Fig. 6G), opposed shortening of colon length (Fig. 6H and I), and colonic inflammation (Fig. 6J). Consistent with the above, lysoPlsEtn treatment suppressed ferroptosis, as indicated by increased GPX4 and decreased COX-2 levels. Together, these results suggest that the presence of the alkenyl-ether group is critical to suppressing colitis and ferroptosis.

## Discussion

Mounting evidence suggests that colitis-associated microbiota shifts were strongly involved in the development of colitis, thereby regulating gut homeostasis [15–17]. In this study, we focused mainly on bacteria-producing ether lipids since these bacteria show specific characteristics for their anaerobic biosynthesis pathways’ possible involvement in the regulation of early-life gut microbiota alteration on colitis. In terms of the gut microbial structure alteration, the development of colitis increased the aerobic bacteria population but reduced the anaerobic bacteria population [18]. Of note, in addition to a reduction in the anaerobic bacteria population, the development of colitis could be induced by high levels of ROS [19]. Given that ether lipids (including plasmalogens) have been proposed to act as ROS scavenger molecules and endogenous antioxidants to govern ferroptosis [8,20,21], we sought to investigate the link between alteration of the gut microbial structure, ferroptosis, and the development of colitis, especially under the different stages of life. Although there is a known association between intestinal inflammation and gut microbiota structure [22,23], the potential mediators of this relationship remain unclear. Here, we presented the characterization of ether lipids-producing anaerobic bacteria in patients or mice with IBDs in early life and observed that the perturbed ether-linked lipid signaling driven by anaerobic bacterial were sufficient for the increased colitis and ferroptosis susceptibility.

Shifts in gut microbiota composition affect various systems, including the development of metabolic, neural, and immune pathways and intestinal homeostasis in early life [24]. Until now, evidence of their direct or indirect impact on intestinal functions has been investigated by other researchers, especially the shift of gut microbiota early in life [25,26]. Previous studies assessing the influence of microbiota depletion or intestinal microbiota modification medicated by antibiotics on colitis in mice yielded conflicting results [27]. Antibiotic therapy, including ciprofloxacin, neomycin, or metronidazole, have been proven to play a preventive role in colitis [28,29]. Similarly, the absence of microbiota observed in germ-free mice and antibiotic-treated mice at 18 weeks of age reduces colonic inflammation, although it impairs barrier function [30]. However, antibiotics exacerbated colitis in interleukin 10-knockout mice at 8 weeks of age [31], and germ-free mice are susceptible to colitis [30,32,33]. Shifts in gut microbiota composition induced by antibiotics also could influence the effect of diet, drugs, or probiotics for colitis prevention [34,35]. These contradictory observations suggested that the age-associated alteration of gut microbiota composition might be involved in the pathogenesis of gut inflammation. However, concerning the association between the age-associated alteration of gut microbiota composition and the pathogenesis of colitis, we observed that early-life microbiota depletion via antibiotic cocktail treatment exacerbated DSS-induced colitis, while mid-life microbiota depletion showed a reduced clinical symptom of colitis upon DSS challenge. Consistent with the observed development of colitis in mice with reduced microbiota at 3 weeks of age by us, early-life microbiota perturbation from days 5 to 10 of life of the pups also showed an increased risk of colitis later in life, which was reported by Blaser et al. [9]. Normally, one possible explanation is that early-life antibiotic-induced perturbation of the microbiome community is considered to decrease the colonic epithelial cell cytoprotective properties of specific bacteria and its specialized metabolites, i.e., short-chain fatty acids or secondary bile acids [36,37]. Although other factors between young and mature adults differ, the different responses to perturbation of microbiome community in young and mature adult mice when treated with DSS indicate a crucial role of the perturbed early-life microbiota in the development of colitis. Our FMT study also showed that restitution of early-life microbiota confers the protection to DSS-induced colitis, although no worsened disease phenotypes were observed post mid-life microbiota transplantation, compared with AIMD mice. Similarly, a recent study also described that restitution of a keystone microbial strain missing in the early-life antibiotic-induced gut dysbiosis results in reduced risk for colitis [38]. Here, we also observed that early-life microbiota dysbiosis renders mice susceptible to ferroptosis. Ferroptosis has recently emerged as one of the causes of IEC death in ulcerative colitis [11]. Indeed, our results indicated that early-life microbiota depletion could exacerbate ferroptosis, while colonization of early-life microbiota confers the suppression of ferroptosis.

The specific bacteria and their metabolites may be involved in the anti-inflammatory effect of early-life microbiota [39–42]. Here, we aimed to identify critical microbes required for the early-life gut microbiome that reduce colitis risk and drives ferroptosis susceptibility. Recently, the genes from microbiomes that contained the pls operon (encodes PlsA and PlsR proteins) were classified as a species as plasmalogen-positive [7], and distribution of the pls operon helped us to assess the distribution of plasmalogen biosynthesis genes. We demonstrated that the young mice exhibit a higher abundance of plasmalogen-positive species in the colon contents than mature adult mice via 16S rRNA sequencing. Because of the limitation of the analysis, we did not accurately quantitate overall plasmalogen molecules in the colon contents of mice. However, by converting plasmalogens to DMA in the presence of acid/methanol, the abundance of DMA derivatives to represent plasmalogens could be analyzed. We found that a higher abundance of plasmalogen-positive species and DMA derivatives in the colonic microbiota community of young mice (3 weeks old) indicated that the plasmalogen-producing microbiota was enriched in the early life of individual development. The above results highlight the relevance of the early-life plasmalogen-positive species and their associated metabolites in intestinal inflammatory conditions. Plasmalogen-positive species from gut microbiota were not only decreased in the mid-life of mice but also reduced in the individuals with colitis (Fig. 4). In a cohort of ulcerative colitis patients and DSS-induced colitis mice, we confirmed the reduction of colonic plasmalogen-producing bacteria abundance and the decrease of plasmalogen levels mirrored by the reduction in the levels of DMA derivatives in the colon contents of DSS-induced colitis mice. In particular, as indicated by extremely lower fecal plasmalogen levels and ether-linked phospholipids levels in microbiota-depleted mice, the presence of colonic ether-link lipids was partly microbiota-dependent. Furthermore, the restitution of ether lipids (plasmalogens) in early life via the oral route promotes suppression of colitis progression in mice at 3 weeks of age, indicating that colonic plasmalogen-producing bacteria may contribute to preventing colitis via its associated metabolite, plasmalogens. Plasmalogens, one kind of phospholipids enriched in the brain and other organs of mammals and in anaerobic bacteria, were thought to be involved in the anti-oxidant and anti-inflammatory function of humans [43]. Besides, decreased plasmalogens seem to be one of the risk factors for inflammatory diseases. Until now, plasmalogen supplementation mainly showed promising health benefits in neurodegenerative diseases and metabolic disorders [44]. Interestingly, we also suggested that plasmalogen treatment exerts anticolitis efficacy in the wild-type mice (Figs. S9 and S10).

Although the role of plasmalogens in inflammatory diseases was reported a long time ago, until now, the function of plasmalogens on intestinal inflammation remained elusive. Recently, plasmalogens were identified from some anaerobic bacteria, including *Bifidobacterium longum* and *Clostridium butyricum* [45,46]. *Bifidobacterium* and *Clostridium butyricum* have been considered probiotics to prevent colitis [47]. However, the biological functions of plasmalogen-positive bacteria to human health remained unknown. In the present study, metronidazole treatment not only resulted in depletion of obligate anaerobic bacteria but also eliminated the presence of plasmalogens as indicated by the entire reduction of DMA levels, which underscored that anaerobic bacterium are the mainly microbial origin for the colonic plasmalogens in mice. Several studies have shown that anti-anaerobic antibiotics pretreatment, including metronidazole and clindamycin, exert intestinal anti-inflammatory effects and alleviate chemically induced colitis and *Citrobacter rodentium*-induced colitis in mice [48–52]. Clinical trials further supported its ability to prevent colitis with modest therapeutic effects [39,53–56]. However, the treatment of clindamycin, one of the antibiotics exceptionally efficient against anaerobic bacteria, promoted the susceptibility to *Campylobacter jejuni*-induced colitis. Importantly, in agreement with other previous studies [9,37], our data supported that depletion of anaerobic bacteria in early life by metronidazole leads to further exacerbated DSS-induced colitis in mice, comparable with those achieved in AIMD mice. Besides, anaerobic microbiota isolated from young mice at 3 weeks of age suppressed intestinal inflammation caused by DSS, further demonstrating the critical role of early-life anaerobic microbiota in the attenuation of colitis progression. To test whether early-life anaerobic microbiota mediates the regulation of intestinal inflammation via plasmalogens, we evaluated the preventive effects of plasmalogens on the progression of colitis in metronidazole-induced anaerobic bacteria-eliminated mice at 3 weeks of age. Similar to colonization with anaerobic microbiota isolated from young mice at 3 weeks of age, plasmalogen treatment suppressed clinical symptom of colitis caused by early-life anaerobic bacteria elimination and showed the protective effects of plasmalogens on the progression of colitis. In terms of the impact of plasmalogens on colitis progression, as expected, plasmalogens attenuate inflammatory responses and revert the enhanced colitis susceptibility of mice lacking anaerobic bacteria.

Analysis of previous reports revealed that microbiota-mediated metabolism could contribute to lipid peroxidation and ferroptosis. Ferroptosis is a novel cell death modality triggered by membrane lipid peroxidation from PUFA-containing phospholipids [8]. PUFA-containing phospholipids are susceptible to peroxidation in oxygen- or iron-rich cellular environments [8]. Cui et al. and Zou et al. [14,57] further showed that ether-linked PUFA-containing phospholipids induce ferroptosis and switch to a ferroptosis-sensitive state. Moreover, it should be noted here that plasmalogens synthesized in humans often contain PUFA at the sn-2 position of the glycerol moiety. However, no PUFA was observed in identified plasmalogen-positive bacterial, such as *Bifidobacterium longum*, *Clostridium difficile*, *Clostridium innocuum*, *Bifidobacterium animalis*, and *Clostridium beijerinckii* [7,43,58–60]. The presence of ether lipids in gut microbiota has been associated with its insensitivity to H_2_O_2_ and confers bacterial survival in intestinal aerobic environments [60,61]. Further evaluation of ether lipids in colitis and ferroptosis will help us to understand the roles of this unique lipid and its linked biological function. Given that PUFA-plasmalogens synthesized in mammalian cells are characterized by the presence of the PUFA chain at position sn-2 and the alkenyl-ether group at position sn-1, although the role of ether-linked lipids in defending against ferroptosis is well established, how microbiota-mediated ether-linked lipids contribute to lipid peroxidation and ferroptosis remains to be investigated. Through data from murine colitis models, we here identified ether lipids as gut microbiota-derived lipids with the potent ability to govern ferroptosis susceptibility. Specifically, ether lipids markedly promoted ferroptosis resistance, and the presence of the alkenyl-ether group is critical to suppressing ferroptosis in colitis. Support our observations, plasmalogens produced by TMEM189 degrade FAR1 and resulted in ferroptosis suppression in OVCAR-8 cells, and plasmalogens exhibited an antiferroptosis role in *Caenorhabditis elegans* and zebrafish [62,63]. Interestingly, the presence of PUFA in PUFA-ether lipids (C18-C20:4 PlsEtn) could gain sensitivity to ferroptosis in OVCAR-8 cells [14] but not duo to the presence of the alkenyl-ether group, which suggested the ether linkage in PUFA-ether lipids does not appear to be important to their function on ferroptosis. Therefore, the role of ether lipids based on the lipid structure, different tissue, and genetic background on ferroptosis came with some discrepancy, which requires further explorations.

Although the role of plasmalogen in inflammatory diseases has long been reported, the function of plasmalogen on intestinal inflammation has not been well defined. Consistent with our findings, dietary ethanolamine plasmalogen could alleviate DSS-induced colitis by enhancing colon mucosa integrity, antioxidative stress, and anti-inflammatory responses [64]. Besides, plasmalogen modulation has been utilized in both preclinical and clinical studies to prevent onset and/or attenuate progression of neurodegenerative diseases and atherosclerosis [65]. However, clinical studies of plasmalogen, especially microbiota-derived ether lipids, on intestinal disease have not been reported. In the present study, we recognized that plasmalogen-producing anaerobic bacteria and their derived ether lipids were involved in the response of early-life microbiota dysbiosis to colitis, and we suggested that ether lipids signaling derived by early-life anaerobic bacteria was sufficient to govern susceptibility to colitis and ferroptosis. Although anaerobic bacteria are not all considered probiotics, even some are pathologic bacteria causing bacterial colitis, we suggested that anaerobic bacteria-mediated lipid metabolism plays a critical in the regulation of colitis and ferroptosis. Of note, gut microbiota-derived ether lipids blocked ferroptosis triggered by gut microbiota dysbiosis and suppressed colitis in mice. Overall, although the mechanisms remain to be elucidated, many lines of evidence pointed out that early-life gut microbiota governs susceptibility to ferroptosis and colitis via ether lipids and suggested the modulation of plasmalogen-positive microbiota as likely targets for intestinal health and treatment of intestinal diseases.

## Methods

### Ethics declarations

The animal study was approved by the Ethical Committees of Jiangnan University under the specific agreement number JN.No2020930c1151121 and JN.No20201230c0200401. Human feces were obtained from patients with IBDs or healthy individuals of First Affiliated Hospital (FAHNMU). Patients or the public were not involved in the design, conduct, reporting, or dissemination plans of our research. It was approved by the Research Ethics Commissions of FAHNMU (2021-SRFA-375) and followed the tenets of the Declaration of Helsinki. Informed consent was obtained from all study participants.

### Human IBD subjects

Feces obtained from patients with IBDs (*n* = 19) or healthy individuals (*n* = 12) were used in this study. Patients with active ulcerative colitis according to accepted clinical and endoscopic criteria were recruited, and the study population included male and female randomized participants of 10 to 30 years of age. Accepted clinical and endoscopic criteria included a Mayo score of ≥5 and ≤9, an endoscopic subscore of at least 2, and a rectal bleeding subscore of at least 1.

### Animals and diets

Male C57BL/6J mice aged 3 and 20 weeks (Weitonglihua, Beijing, China) were used in compliance with the ARRIVE guidelines. Mice were raised in an SPF environment with free access to water and food (AIM-93G). Animals were submitted to standard 12-h light/dark cycles. Plasmalogens (PlsEtn) were extracted according to our previous method [44]. Lysoplasmenylethanolamine were prepared via mild alkaline methanolysis according to the previous method of Hanahan et al. [66].

### DSS model of colitis and bacterial culture

Colitis was induced by DSS (Meiluobio, China). Animal allocation to treatment groups was randomized. For induction of acute experimental colitis, mice received 7 days of 3% DSS dissolved in drinking water and followed by 3 days of regular drinking water. Prior to the acute DSS-induced colitis, in a cohort of 3-week-old and 20-week-old mice, a cocktail of 4 antibiotics (ampicillin [25 mg/kg], vancomycin [12.5 mg/kg], metronidazole [25 mg/kg], and neomycin (25 mg/kg]) or sterile saline was gavaged to mice at 200 μl per mouse once every day for 7 days to deplete their gut microbiota. After that, AIMD mice received 7 days of 3% DSS or regular drinking water and were gavaged by plasmenylethanolamine or lysoplasmenylethanolamine (100 mg/kg body weight) or sterile saline.

Another cohort of 3-week-old mice received metronidazole (25 mg/kg) was gavaged to mice at 200 μl per mouse once every day for 7 days to deplete their anaerobic bacteria. After that, metronidazole-induced anaerobic bacteria-depleted mice received 7 days of 3% DSS or regular drinking water. The stool of 3-week-old and 20-week-old mice was collected as conventionalized microbiota of young and adult mice, respectively. Freshly collected stools were homogenized in phosphate-buffered saline (0.05% cysteine HCl) at a ratio of 5 fecal pellets/ml. For FMT, the fecal slurry from 3-week-old and 20-week-old mice was delivered to the AIMD mice (Young-FMT and Adult-FMT) by oral gavage (200 μl) every second day. Given the coprophagic nature of rodents, these mice were single-housed to exclude potential confounding impacts including the cage effect. During this time, the mice received 7 days of 3% DSS or regular drinking water. Using brain hart infusion or Luria broth agar plates, freshly collected stools from 3-week-old mice were cultured under aerobic, microaerobic, or anaerobic conditions. AIMD mice were orally gavaged with the microbiota cultured above (1 × 10^8^ CFU, every second day). These mice also received 3% DSS or regular drinking water. Mice were weighed daily and visually inspected for diarrhea (stool consistency) and rectal bleeding. Among them, rectal bleeding was scored as 0, 2, and 4 (normal, slight bleeding, and gross bleeding, respectively). Diarrhea was scored as 0, 2, and 4 (normal, loose stools, and watery diarrhea, respectively).

### Cell culture

Human HT-29 colon carcinoma cells (HT-29) (Shanghai, China) were cultured with minimum essential medium containing 10% fetal bovine serum. HT-29 cells were pretreated with arachidonic acid (100 μM) for 24 h. Cell viability were measured in lyso-PlsEtn (50 μg/ml, 16 h) or ML210-treated (GPX4 inhibitor, 5 μM, 16 h) HT-29 cells.

### Analysis of lipids

Collected colon contents of mice were freeze-dried, and whole lipids were extracted as the methods of Sugawa et al. [67]. The identification of DMA and FAME was analyzed by gas chromatography–mass spectrometry analysis [7].

### Bacterial community analysis

Total DNA was extracted from feces, and then bacterial community analysis was performed as previously done by GENEWIZ, Inc. (Suzhou, China) [68]. As for plasmalogen-positive microbiota analysis, plasmalogen-positive microbiota species were provided as the results of Jackson et al. [7].

### Real-time polymerase chain reaction

Total RNA was extracted and reverse transcribed using TRIzol reagent and cDNA Synthesis SuperMix (Yeasen Biotech, China). Real-time polymerase chain reaction was carried out in Applied Biosystems QuantStudio 3 with Hieff qPCR SYBR Green master mix (Yeasen Biotech, China) and gene-specific primers.

### Statistical analysis

All results are expressed as means ± SEM. Computations assumed that all groups were samples from populations with the same scatter. Statistical significance was determined by Student *t* test or 2-way analysis of variance. The probability of *P* value < 0.05 was indicated a significant difference.

## Data Availability

The data are available from the author (Yanjun Liu) upon reasonable request.

## Supplementary Materials

Supplementary Materials Fig. S1. Culture-dependent and culture-independent analysis confirmed that the fecal microbiota or anaerobic bacteria were depleted in AIMD mice or metronidazole-induced anaerobic bacteria-eliminated mice. Fig. S2. Ferroptosis was triggered in DSS-induced colitis. Fig. S3. Early-life microbiota depletion induced lipid peroxidation. (A and B) Colonic MDA levels and (C and D) 4-HNE levels were measured. Fig. S4. Immunofluorescence analysis of GPX4. Fig. S5. Fecal plasmalogens and ether-linked phospholipids levels were reduced in mice with microbiota depletion. Fig. S6. The abundance of DMA in colon contents of control and metronidazole-induced anaerobic bacteria-eliminated mice. Fig. S7. Early-life anaerobic bacteria elimination exacerbates DSS-induced colitis. Fig. S8. Intestinal anaerobic bacteria in early life protect against colitis. Fig. S9. Plasmalogens suppress DSS-induced acute colitis in mice. Fig. S10. Plasmalogens suppress DSS-induced acute colitis in adult mice. Fig. S11. Plasmalogens reverts the aggravated intestinal inflammation caused by anaerobic bacteria elimination. Fig. S12. Lysoplasmenylethanolamine alleviated arachidonic acid-induced HT29 cell damage.
