# Supplementary material for: Early-Life Gut Microbiota Governs Susceptibility to Colitis via Microbial-Derived Ether Lipids
Source: Research (Wash D C). 2023 Jan 13;6:0037. doi: 10.34133/research.0037 (PMC10076029; doi:10.34133/research.0037)
Supplement: Supplementary Materials — Fig. S1. Culture-dependent and culture-independent analysis confirmed that the fecal microbiota or anaerobic bacteria were depleted in AIMD mice or metronidazole-induced anaerobic bacteria-eliminated mice. Fig. S2. Ferroptosis was triggered in DSS-induced colitis. Fig. S3. Early-life microbiota depletion induced lipid peroxidation. (A and B) Colonic MDA levels and (C and D) 4-HNE levels were measured. Fig. S4. Immunofluorescence analysis of GPX4. Fig. S5. Fecal plasmalogens and ether-linked phospholipids levels were reduced in mice with microbiota depletion. Fig. S6. The abundance of DMA in colon contents of control and metronidazole-induced anaerobic bacteria-eliminated mice. Fig. S7. Early-life anaerobic bacteria elimination exacerbates DSS-induced colitis. Fig. S8. Intestinal anaerobic bacteria in early life protect against colitis. Fig. S9. Plasmalogens suppress DSS-induced acute colitis in mice. Fig. S10. Plasmalogens suppress DSS-induced acute colitis in adult mice. Fig. S11. Plasmalogens reverts the aggravated intestinal inflammation caused by anaerobic bacteria elimination. Fig. S12. Lysoplasmenylethanolamine alleviated arachidonic acid-induced HT29 cell damage. [file research.0037.f1.docx]

**Supplementary Materials**

Figure S1: culture-dependent and culture-independent analysis confirmed that the fecal microbiota or anaerobic bacteria were depleted in antibiotic-induced microbiota depleted mice or metronidazole-induced anaerobic bacteria-eliminated mice. (a) Representative images of LB and BHI plating of feces from antibiotic cocktail- and metronidazole-treated mice. (b) Bacterial DNA was absent from fecal samples collected from AIMD mice. Data are shown as individual points with mean ± SEM. n = 6 mice per group. ** *P <* 0.01. Figure S2: [ferroptosis](https://www.sciencedirect.com/topics/medicine-and-dentistry/ferroptosis) was triggered in DSS-induced colitis. (a) Body weight change, (b) Colon length, (c) colonic total iron levels, (d) MDA levels were measured in DSS-induced colitis. (e) Colon tissues were examined histologically after H&E staining (scale bars, 100 μm). (f, g, h, i) Representative immunoblots and quantitative analysis of ferroptosis-related proteins (COX-2, GPX4, ACSL4). (j, k, l) representative IHC staining and quantitative analysis of COX-2 and GPX4. (m, n, o, p) Relative mRNA expression of *Cox-2*, *Acsl4*, *Gpx4*, and *Fth1* in colon were checked. Figure S3: early-life microbiota depletion induced lipid peroxidation. (a, b) Colonic MDA levels and (c, d) 4-HNE levels were measured. Figure S4: Immunofluorescence analysis of GPX4. Figure S5: fecal plasmalogens and ether-linked phospholipids levels were reduced in mice with microbiota depletion. (a) DMA levels, (b) ether-linked phospholipids levels were measured. Figure S6: the abundance of DMA in colon contents of control and metronidazole-induced anaerobic bacteria-eliminated mice. Figure S7: early-life anaerobic bacteria elimination exacerbates DSS-induced colitis. (a) Study design using male metronidazole-induced anaerobic bacteria-eliminated mice at the age of 3 weeks. 3-week-old mice were subjected to a one-week oral administration of metronidazole and then treated with 3% DSS for seven days. (b, c) Stools consistency and colorectal bleeding were scored in metronidazole-treated or untreated mice. (d) The body weight of metronidazole-treated or untreated mice was measured daily. (e, f) Survival curve of metronidazole-treated or untreated mice following treatment with 3% DSS. (g) Colon tissues of metronidazole-treated or untreated mice were examined histologically after H&E staining (scale bars, 100 μm). (h, i) Metronidazole-treated or untreated mice were euthanized on day 17, and colon lengths were measured. (j, k) Heatmap showing the relative expression levels of genes involved in the inflammatory response in metronidazole-treated or untreated mice. Figure S8: intestinal anaerobic bacteria in early life protect against colitis. (a) Study design using microbiota colonization. Young normobiotic mice (3-week-old) were treated with a broad-spectrum antibiotic cocktail and were then colonized with the respective microbiota (cultured under aerobic, microaerobic or anaerobic conditions), and we treated the mice with 3% DSS for seven days. (b) The body weight of mice colonized with the respective microbiota (Aero, MicroAero, and Anaero) was measured daily. (c, d) Stools consistency and colorectal bleeding were scored in mice colonized with the respective microbiota (Aero, MicroAero, and Anaero). (e, f) Mice colonized with the individual microbiota (Aero, MicroAero, and Anaero) were euthanized, and colon lengths were measured. (g) Colon tissues of mice colonized with the respective microbiota (Aero, MicroAero, and Anaero) were examined histologically after H&E staining (scale bars, 100 μm). Figure S9: plasmalogens suppress DSS-induced acute colitis in mice. (a) Experimental approach. Mice at 3-weeks of age were treated with plasmalogens through an oral route. Plasmalogens-treated or vehicle-treated mice were fed with 3% DSS for seven days. (b) The body weight of plasmalogens-treated or vehicle-treated mice was measured daily. (c, d) plasmalogens-treated or vehicle-treated mice were euthanized, and colon lengths were measured. (e, f) Colorectal bleeding and Stools consistency were scored in plasmalogens-treated or vehicle-treated mice. (g) Colon tissues of plasmalogens-treated or vehicle-treated mice were examined histologically after H&E staining (scale bars, 100 μm). Figure S10: plasmalogens suppress DSS-induced acute colitis in adult mice. Mice at 20-weeks of age were treated with plasmalogens through an oral route. Plasmalogens-treated or vehicle-treated mice were fed with 3% DSS for seven days. (a) The body weight of plasmalogens-treated or vehicle-treated mice was measured daily. (b, c) Colorectal bleeding and stools consistency were scored. (d) Colon lengths were measured. Figure S11: plasmalogens reverts the aggravated intestinal inflammation caused by anaerobic bacteria elimination. (a) Experimental approach. Mice at 3-weeks of age were subjected to a one-week oral administration of metronidazole and then treated with plasmalogens and given 3% DSS in drinking water for seven days. (b) The body weight of plasmalogens-treated or vehicle-treated mice (anaerobic bacteria eliminated) was measured daily. (c) Survival curve of mice. (d, e) plasmalogens-treated or vehicle-treated mice (anaerobic bacteria eliminated) were euthanized, and colon lengths were measured. (f, g) Stools consistency and colorectal bleeding were scored in plasmalogens-treated or vehicle-treated mice (anaerobic bacteria eliminated). (h) Heatmap showing the relative expression levels of genes involved in the inflammatory response in plasmalogens-treated or untreated mice (anaerobic bacteria eliminated). (i) Colon tissues of plasmalogens-treated or vehicle-treated mice (anaerobic bacteria eliminated) were examined histologically after H&E staining (scale bars, 100 μm). (j) Immunofluorescence staining revealed co-localization of iNos (green) and CD163 (red) expression and quantification of iNos and Cd163. Figure S12: Lysoplasmenylethanolamine alleviated arachidonic acid-induced HT29 cell damage.
